# Carbon nanoparticles with oligonucleotide probes for a label-free sensitive antibiotic residues detection based on competitive analysis

**DOI:** 10.1038/s41598-019-40209-1

**Published:** 2019-03-05

**Authors:** Xuexia Lin, Jianlong Su, Honggui Lin, Shu-Feng Zhou, Xiangying Sun, Bin Liu, Mingrong Zeng

**Affiliations:** 1 Depaertment of Chemical Engineering & Pharmaceutical Engineering, College of Chemical Engineering, Huaqiao University, Xiamen, 361021 China; 2 School of Marine Engineering, Jimei University, Xiamen, 361021 China; 3 College of Materials Science and Engineering, Huaqiao University, Xiamen, 361021 China

## Abstract

Carbon nanoparticles (CNPs) have been combined with aptamer, providing a broad application in small molecule. CNPs can be quenched by small molecules and are usually applied as luminescent probes because of their photophysical characteristics. In this work, we developed a competitive analysis for antibiotic residues detection based on carbon nanoparticles (CNPs) and oligonucleotide probes. Oligonucleotide probes including oxytetracycline (OTC) aptamer was exploited for recognition OTC and was used to restore the luminescence. Tetracycline (TC), as a competitor of OTC, was utilized to quench the luminescence of CNPs and reduce the sample matrix effect. Under optimal conditions, the linear rang of OTC was 0.010~1.0 ng/mL with the relative standard deviations (RSDs) from 2.91% to 11.3%, and the limit of detection (LOD) was low to 0.002 ng/mL. Moreover, the proposal was successfully applied to analyze OTC from drink water, indicating that this approach has great potential for other small molecule analysis.

## Introduction

Advances in nanotechnology development, multifunctional nanoparticles have been performed various functions including targeting, imaging technology, and therapy. Gold nanoparticles, silver nanoparticles, silica nanoparticles and carbon nanomaterials have been investigated for widespread applications as sensors, therapeutic agents, drug delivery, optical imaging and so on. Recently, carbon nanomaterials such as CNPs have received lots of attention due to their unique properties including self-illumination, less toxicity, versatile surface modification, green synthetic route, optical stability, and good bio-compatibility. Importantly, it was found that CNPs have strong luminescence in the visible and near-infrared spectral regions with photochemically stability and nonblinking charactristic, which is greatly different from the semiconductor quantum dots^1,2^. Therefore, they have been considered as new sensors for applications in small molecule assay, biosensors, bioimaging, cancer therapy, and optosensors. X. Chen group designed and prepared chlorin e6-conjugated CNPs as the light-triggered theranostics for simultaneous enhanced photosensitizer fluorescence detection and photodynamic therapy based on Förster resonance energy transfer (FRET) mechanism^3^. In early work, CNPs with both one- and multiple-photon excitations have been developed for fluorescence imaging of cells and tissues^4–6^. Recently, some groups have exploited CNPs to label cell organ not to label the whole cell^7,8]^. Moreover, CNPs have been widely used in various sensors^9,10^. Jin-Ming Lin group utilized CNPs to enhance chemiluminescence^11,12^, and D.-G. Ma *et al*. made up white light-emitting devices based on CNPs’ electroluminescence^13^.

Nucleic acid aptamers are highly structured oligonucleotides screened from DNA/RNA libraries. With high specificity and affinity, aptamers are considered to be used to replace antibodies and are widely used in various fields^14,15^. More importantly, aptamers can be modified and combine with a variety of nanoparticles to form new sensors in environmental monitoring, intracellular monitoring, therapeutic applications and biotechnology^16–18^. Because single strand DNA(ssDNA) can be adsorbed on CNPs by π-π stacking to result in quenching of fluorescence by FRET, Li *et al*. have developed a fluorescence sensing platform for nucleic acid detection^19^. Due to the high specificity of aptamers, C. J. Yang group has reported that a highly sensitive detection of ATP and kanamycin based on the protective properties of CNPs to ssDNA from enzymatic cleavage^20^. More analytical platforms have been developed for the detection of DNA, proteins, nucleases and metal ions based on CNPs and aptamer. Therefore, it is possible to develop a convenient and sensitive assay based on aptamer and CNPs.

**Figure 1:**
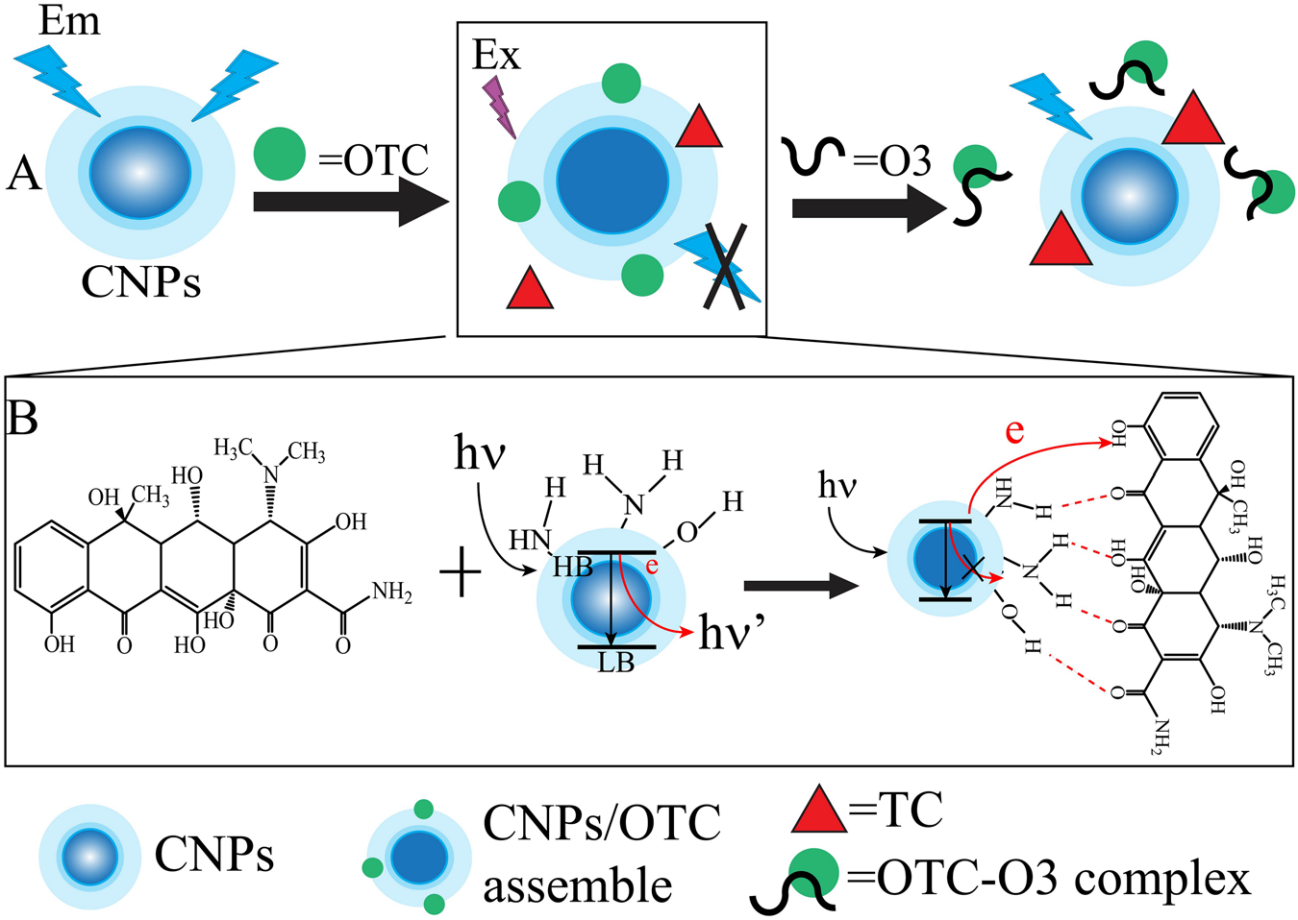
(**A**) The illustration of competitive analysis for OTC based on aptamer and CNPs. (**B**) The mechanism of OTC inducing quenching of CNPs.

Herein, we developed a rapid and sensitive method for the determination of antibiotic residues from drinking water based on the application of CNPs and ssDNA. In this work, CNPs serve as a promising luminescent sensor because antibiotic residues cause the quenching luminescence of CNPs in Fig. 1. Aptamers can be bond to antibiotic residue and enable to make antibiotic residues keep away from CNPs, leading to the recovery of luminescence. OTC was selected as a model analyte since it has been extensively used as an antibiotic and growth promoter. The accumulation in food products and environment gives rise of serious risks to human health. TC was used as a quencher and as a competitor. Due to aptamers with high affinity and specificity, the proposal would excellent selectivity and the high sensitivity is resulted from the application of TC.

## Results

### The synthesis, characterization and photophysical properties of CNPs

**Figure 2:**
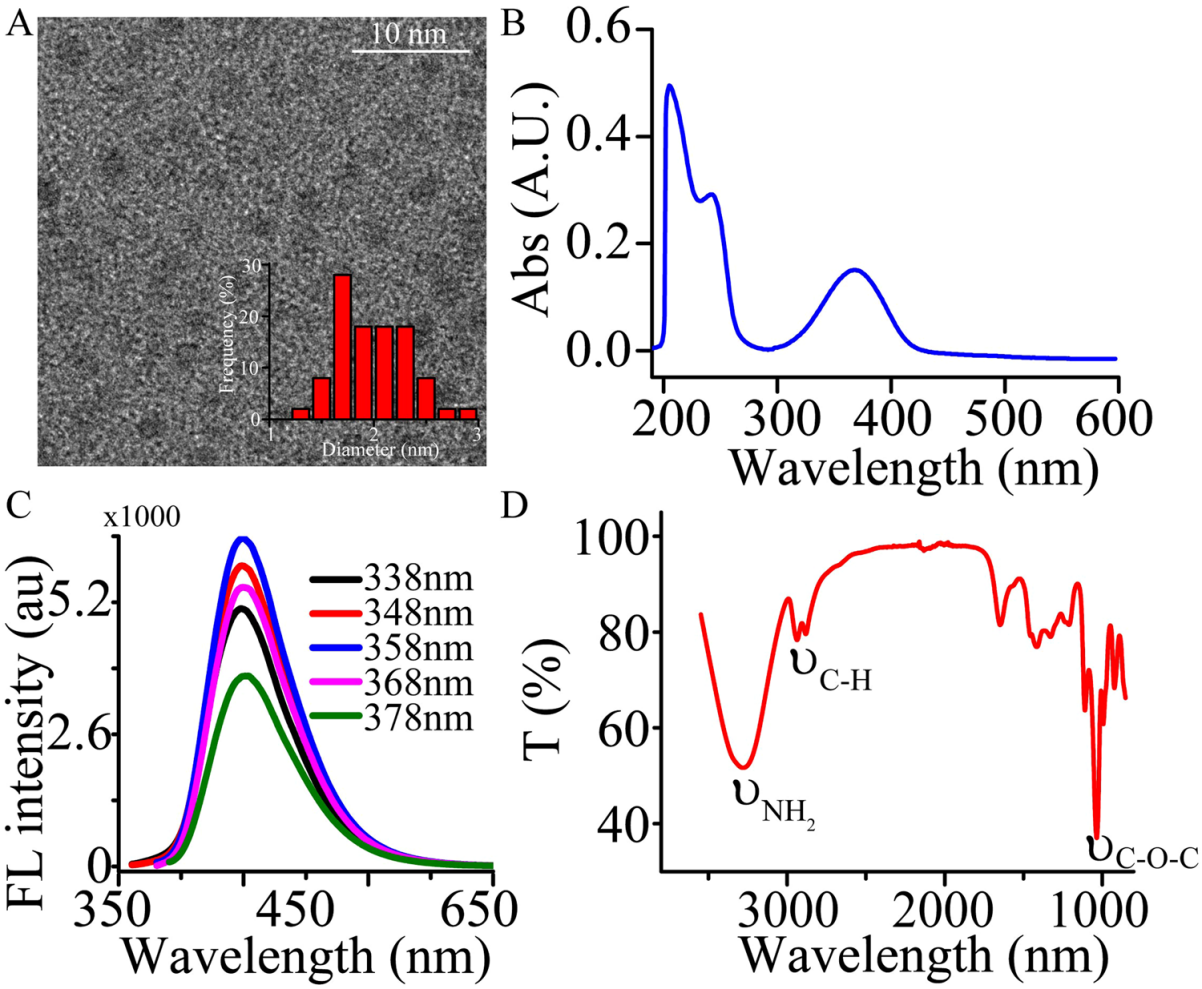
(**A**) TEM images of the CNPs. The inset shows the particle size distribution histograms of CNPs. (**B**) UV-vis absorption spectrum of CNPs. (**C**) Photoluminescence emission spectra of the CNPs obtained at different excitation wavelengths with a 10 nm increment from 338 to 378 nm. (**D**) FTIR spectrum of CNPs. A.U. means absorbance unit. au means arbitrary unit.

CNPs were synthesized by microwave method, and glycerine and ethylenediamine were used as both carbon source and passivating agent. As shown in Fig. 2A, according to TEM observation, the sample consists of well-dispersed nanoparticles, and the statistical particle size distribution of CNPs ranged from 1 nm to 3 nm. The major formation path of the CNPs is the dehydration and carbonization mechanism during microwave synthesis. In addition, lots of peptide bond can be obtained, which can react with the carbon chain of the glycerin. UV-vis spectra displays strong absorption band from 200 nm to 275 nm in Fig. 2B, which can be attributed to π–π* transition of C=C, and a peak at approximately 358 nm, which can be attributed to n–π* transition of C=O. As the excitation wavelength increases (338 nm~378 nm), the emission doesn’t shift although the intensity changes in Fig. 2C. The maximum emission peak is at λ = 452 nm with an excitation wavelength of 358 nm. It also can be observed that the change of pH value (ranging from 6.2 to 8.5) has little affected on the intensity in Fig. S1.

FTIR spectra shows an absorption peak of the-NH_2_ group at 3285 cm^−1^ and an absorption peak of the C=O group conjugated with condensed aromatic carbons at 1647 cm^−1^ in Fig. 2D. These reveal the existence of carboxylate and amine groups on the surfaces of the CNPs. The data indicated that the obtained CPNs are rich in carboxylic and amine groups. The sharp peak at 2937 cm^−1^ and 2882 cm^−1^ are attributable to the methylene groups in the long alkyl chain. A sharp band at 1035 cm^−1^, corresponding to the C-O-C asymmetric stretching vibration, was observed. The peak of 1398 and 1415 cm^−1^ indicated that the asymmetric stretching vibration of the carboxylate anions and the symmetric stretching vibrations of the carboxylate anions were detected. The absorption peaks at 992, 1045, 1079, and 1109 cm^−1^ were ascribed to C-O stretching vibrations, suggesting that the surfaces of the C-dots are oxygen-rich.

### Designing of ssDNA

**Figure 3:**
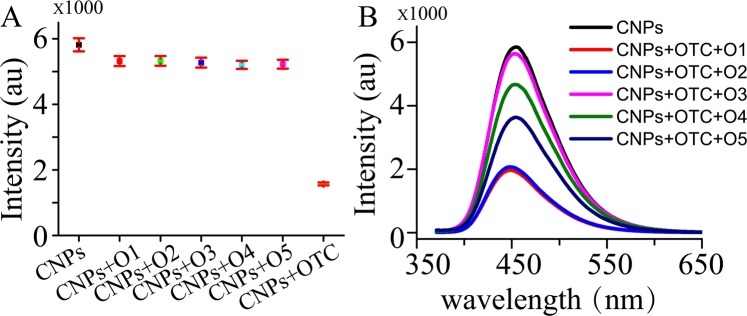
Optimization of ssDNA without OTC (**A**) and with OTC (**B**). Each error bar is obtained by data analysis of three parallel experiments.

The luminescence response of CNPs upon addition of different concentrations of OTC or TC was first investigated by emission titration experiments. Figure S2 shows that OTC and TC can interact with CNPs, inducing quenching of CNPs. Moreover, the luminescence of CNPs is significantly reduced in the presence of the increasing concentration of OTC than that of TC. Encouraged by quenching luminescence of CNPs, the distinctive sensing behavior of CNPs to OTC and ssDNA was studied. Several kinds of ssDNA (O1, O2, O3, O4 and O5) containing OTC aptamer were designed in Table 1. Figure 3A shows with the addition of ssDNA, the luminescence intensities of the system containing OTC are strongly enhanced while the intensities are almost consistent with that of CNPs without OTC and each experiment was done three times in parallel. Therefore, the possible sensing mechanism of OTC detection is proposed in Fig. 1. TC and OTC were used to quench the luminescence of CNPs, and ssDNA contained OTC aptamer was applied to the luminescent recovery because OTC can bond to ssDNA and keep away from CNPs. Under this hypothesis, ssDNA was designed and optimized. Although O4 and O5 also can restore the luminescence, the application of O3 can be in a strongest enhancement of the luminescence in Fig. 3B. However, the addition of ssDNA O1 or O2 did not restore the signal. As depicted in Fig. S3, when the utility of O3 and O4, the luminescence was gradually enhanced with increasing the concentration of OTC, and stronger luminescence was enhanced by exploiting O3. Besides, when O5 was present, the luminescence was little changed with increasing OTC. These demonstrated that O3 is more effective for OTC analysis compared to the system using O4 and O5.

**Table 1:** Various kinds of ssDNA used in this study.

ssDNA name	ssDNA size	ssDNA sequence(5′-3′)
O1	74	TTTGGAATTCGCTAGCACGTTGACGCTGGTGCCCGGTTGTGGTGCGAGTGTTGTGTGGATCCGAGCTCCACGTG
O2	77	TTTTTTGGAATTCGCTAGCACGTTGACGCTGGTGCCCGGTTGTGGTGCGAGTGTTGTGTGGATCCGAGCTCCACGTG
O3	80	TTTTTTTTTGGAATTCGCTAGCACGTTGACGCTGGTGCCCGGTTGTGGTGCGAGTGTTGTGTGGATCCGAGCTCCACGTG
O4	83	TTTTTTTTTTTTGGAATTCGCTAGCACGTTGACGCTGGTGCCCGGTTGTGGTGCGAGTGTTGTGTGGATCCGAGCTCCACGTG
O5	85	TTTTTTTTTTTTTTTGGAATTCGCTAGCACGTTGACGCTGGTGCCCGGTTGTGGTGCGAGTGTTGTGTGGATCCGAGCTCCACGTG

### Developing OTC detection method

**Figure 4:**
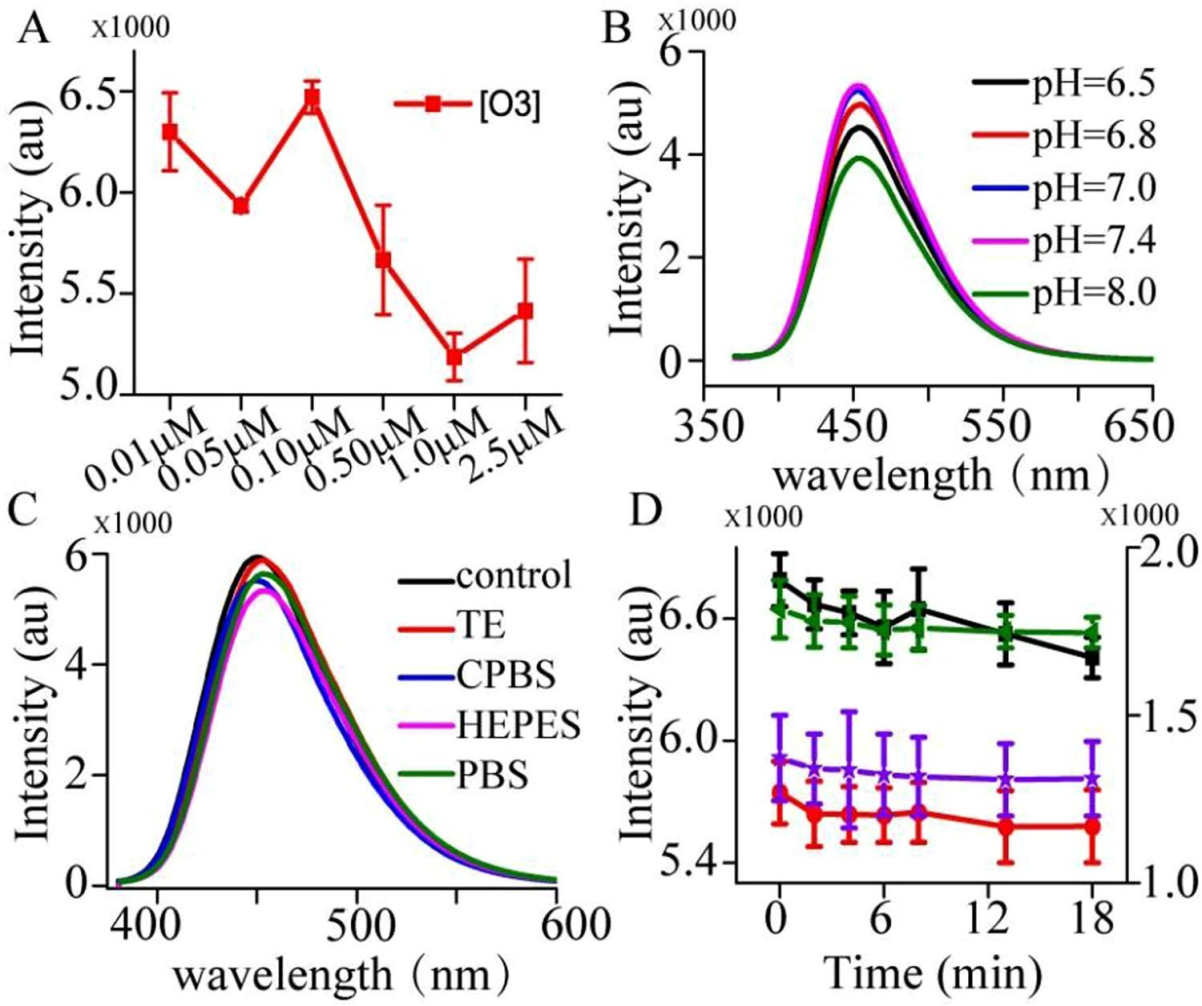
Optimization conditions for OTC analysis. (**A**) The concentration of ssDNA O3. (**B**) pH value. (**C**) The kinds of buffer. (**D**) The time-dependent luminescent changes upon analyzing different concentrations of OTC. Each error bar is obtained by data analysis of three parallel experiments.

To optimize the performance of the system, we initially investigated the effect of TC, and various concentrations of TC were studied. Figure S2 shows that the intensities are gradually decreased with the increasing TC upto 25 ng/mL with 10 μg/mL CNPs. Furthermore, the concentration of ssDNA O3 was also studied in the presence of OTC. The results reveals that 0.1 μM of aminated ssDNA O3 is optimal for OTC assay in Fig. 4A, as higher concentrations gives a high background, while lower concentration causes reduction of sensitivity. In contrast, no enhancement in emission intensity was observed upon addition of O3 in absence of OTC. Under the optimal concentration of O3, we investigated pH value of Tris-EDTA (TE, 50 mM Tris, 1 mM EDTA) buffer and pH value was ranged from 6.5 to 8.5 by hydrochloric acid solution. Figure 4B depicts that the strongest luminescence response is achieved at pH = 7.0/7.4, while it is weak at other pH values. Because drink water often contain some metal ions, different kinds of buffer including TE buffer, 0.01 M phosphate buffer saline (PBS), 1.0 M 4-(2-hydroxyethyl)-1-piperazineethanesulfonic acid (HEPES) buffer, and 0.2 M citrate phosphate buffer (CPBS) were studied to reduce the matrix effect. Figure 4C shows that the intensity of the system by using TE buffer is higher than others. Therefore, TE buffer with pH = 7.4 was used in the next experiment. The detection time was also optimized. From Fig. 4D, it can be found that the intensity is gradually decreased with increasing time upto 10 min, and then maintained until to 18 min. This indicated that the reaction balance was achieved ranging from 10 min to 18 min. In order to realize rapid analysis, 10 min was taken for the next experiment

### Selectivity and sensitivity of OTC detection

**Figure 5:**
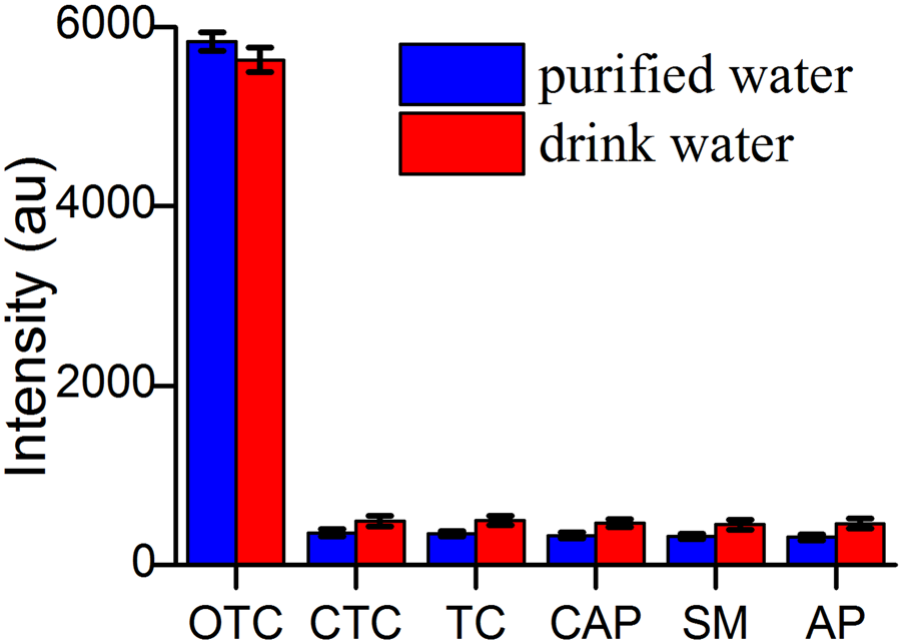
The selectivity of ssDNA for OTC assay based on CNPs. The concentration of OTC was 1.0 pg/mL while the others were 10 ng/mL. CTC, TC, CAP, SM. AP indicate chlortetracyclin, tetracycline, chloramphenicol, streptomycin and ampicillin, respectively. Each experiment was done three times in parallel.

To verify the selectivity of the proposal, chlortetracyclin (CTC), tetracycline (TC), chloramphenicol (CAP), streptomycin (SM) and ampicillin (AP) have been used, and were respectively spiked into water sample with the concentration of 10 ng/mL. As shown in Fig. 5, under optimal conditions, the luminescence of OTC (1.0 ng/mL) is higher than others. Although the concentration of OTC (1.0 ng/mL) is only 1/10 of that (10.0 ng/mL) in the control group, demonstrating this system has an excellent selectivity. In order to study the selectivity of the system in the complex water environment, OTC was 1.0 ng/mL and other antibiotics were the concentration of 10.0 ng/mL. They were spiked into drink water sample, respectively. The results exhibited great selectivity of the system, indicating the potential application of our proposal in real sample analysis.

**Figure 6:**
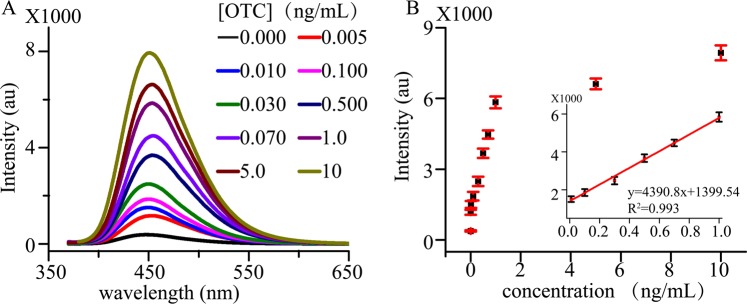
Quantitative analysis for OTC. (**A**) Luminescence spectra of different concentrations of OTC. (**B**) The intensity of the system with different concentrations of OTC. Inset shows a linear range from 0.010 ng/mL to 1.0 ng/mL. Each error bar is obtained by data analysis of three parallel experiments.

Under the optimal conditions, the system exhibited the strongest emission band centered at 452 nm when excited at 358 nm. To achieve quantitative analysis of OTC, a series of OTC standard solutions were determined. Figure 6A shows the fluorescence spectra and intensities of OTC (0.005~10.0 ng/mL) analyzed with the proposal. An excellent linear correlation of y = 4390.86x + 1399.54 (R^2^ = 0.993) in the range from 0.01 ng/mL to 1.00 ng/mL was achieved in Fig. 6B. The quantitative analysis of OTC has a good reproduction with RSDs between 1.27% and 7.39%. LOD defined by signal-to-noise of 3 was as low as 0.002 ng/mL (∼0.0043 nM, given the molecular weight of OTC is 460.43). The sensitivity of the established method is more sensitive or comparable to that of the other methods such as fluorescent and electrochemical techniques (Table S1). These results demonstrated that the established method can be applied to detect OTC with high sensitivity and excellent selectivity.

### OTC detection in drink water sample

We chose drink water as analytes to show that the assay is applicable for real sample analysis. Since drink water contains kinds of small molecule such as metal ion which might interfere with OTC adsorbed on CNPs, it is difficult to detect OTC in non-treated drink water. Therefore, we investigated drink water treated with TE buffer. The pH value of drink water was fixed at 7.4. As shown in Table 2, seven kinds of drink water were analyzed. There was not detect OTC in seven kinds of drink water, and two concentrations (1.0 ng/mL and 0.010 ng/mL) of OTC were spiked into drink water by the standard addition method and were determined by the established method. The recoveries changed from 86% to 93% with the RSDs ranging from 2.91% and 9.41% (n = 3). The results suggest that the developed method is feasible. Even better, only about 10 min was needed in the whole analytic process. Thus, this work provides a simple, rapid, and low cost strategy to analyze OTC, which was potential for water environmental analysis.

**Table 2:** OTC was analyzed by the developed method and the recovery of OTC spiked into drink water samples (n = 3).

Sample No.	Detection concentration (ng/mL)	Spiked into drink water (ng/mL)	Detection concentration (ng/mL)	Recovery (%)	RSD (%)	Spiked into drink water (ng/mL)	Detection concentration(ng/mL)	Recovery (%)	RSD (%)
1	—	0.010	0.0090	90.0	2.91	1.0	0.99	99	6.72
2	—	0.010	0.0086	86.0	6.32	1.0	0.89	89	5.23
3	—	0.010	0.0093	93.0	6.21	1.0	0.92	92	3.92
4	—	0.010	0.0097	97.0	3.76	1.0	0.96	96	5.76
5	—	0.010	0.0094	94.0	5.36	1.0	0.94	94	4.21
6	—	0.010	0.0088	88.0	9.41	1.0	0.92	92	4.79
7	—	0.010	0.0090	90.0	8.58	1.0	0.93	93	3.26

## Discussion

FT-IR spectra were used to identify the surface chemical groups presenting on the prepared CNPs in Fig. 2. It reveals that the surface of CNPs are oxygen-rich, and the presence of –OH, C–H, –COOH, C=O, C–O–C, C–O and -NH_2_. These functional groups are hydrophilic, which improve the water solubility and stability of CNPs. The remarkable optical properties of the CNPs were confirmed by UV-vis absorption and fluorescence spectra. The emission spectrum of the CNPs showed a narrow and symmetrical peak centered at 452 nm when the excitation wavelength was at 358 nm. The luminescence intensity of the CNPs was almost invariable as the influences of pH value, demonstrated that the prepared CNPs have relative stability and excellent photostability. These results hint that CNPs have outstanding stability in ambient environment and can be employed as a luminescent probe.

Since the more significantly reduction of photoluminescence of OTC than that of TC in Fig. S2, it is possible to use TC as quenchers and competitors for the development of a label-free OTC detection. Furthermore, TC is an analogue of OTC. According to these, we designed a strategy as shown in Fig. 1. In this work, the surface of synthesize CNPs contain amino groups and hydroxyl group, which can bind to OTC or TC. When OTC was combined with CNPs, the surface of CNPs was changed and the luminescence was quenched. We deduced that the photoluminescence of the CNPs is caused by the few electrons in the high energy band for the CNPs at absolute zero and holes on the surface according to the refs^21,22^. In the present of OTC, OTC can accept the excited-state electron, leading to the failure of the recombination of electron and hole on the surface of CNPs. This recombination failure would cause fluorescence quenching. CNPs have been reacted with OTC and TC. Unbound TC in the solution can react with CNPs when OTC on the CNPs was removed, leading to a reliable control background and the low limit of detection. To test whether ssDNA alone would have influence on the luminescence of CNPs, we repeated the experiment in the absence of OTC. The luminescence intensity of CNPs was little affected in Fig. 3A. Nevertheless, if OTC is present, the luminescent signal is partial recovered. We deduced that OTC bind with ssDNA O3 and are away from CNPs, resulting in the restore. So, we presumed CNPs can be partially protect by ssDNA, and explained that the enhancement of the system luminescence is likely due to the release of OTC-induced quenching by ssDNA.

Different sizes of ssDNA were examined by the emission titration under the optimal conditions. The results demonstrated that lengthening the oligonucleotide (O3 and O4) would ensure good binding sites for OTC and promote OTC to keep away from CNPs. The lengthening of ssDNA is also possibly strengthening the binding of OTC to ssDNA and augmenting signal enhancement. O3 is more effective for OTC analysis compared to the system using O4 and O5, demonstrating too long ssDNA (O5) leading to an inability to luminescence respond maybe due to the enclose CNPs and OTC by ssDNA. A high background was observed when the concentration of O3 was higher than 0.1 μM, while lower concentrations caused reduction of sensitivity. These not only illustrated that O3 was optimal, but also demonstrated that the balance among CNPs, OTC and O3 can be rapidly achieved when 0.1 μM O3 was used.

pH value is important for the structure of OTC. It also has effect on the reaction of O3, OTC and CNPs. The influence of pH on the luminescence intensity was investigated in the range of pH 6.5~8.5, and it is found that the strongest luminescence response was achieved at pH = 7.4 in Fig. 4B. These indicated that O3 and CNPs can well work in the system for the determination of OTC at 50 mM TE buffer with pH = 7.4. In this work, we found that TE as buffer is better than other buffer such as HEPES, PBS and CPBS. We deduced that TE buffer contained EDTA, which can chelate metal ions from drink water itself. Moreover, the time-dependent luminous intensity upon different concentrations of OTC demonstrated that the balance of the system was achieved at 10 min. It also illustrated that the detection time can be reduced to 10 min.

A study on the platform to distinguish OTC was also performed. The OTC aptamer was used since CNPs is not a dedicated probe for oxytetracycline. The other antibiotics were exploited to study the selectivity of platform. The luminescence signal of the platform that is the combined use of CNPs and O3 did not recover upon addition of other antibiotics. This illustrated that O3 can effectively bind to OTC and our established platform has excellent selectivity. Based on this point, we performed an experiment in the presence of CNPs and O3 with the concentration of OTC ranging from 0.005 to 10 ng/mL. The linear range of OTC extends from 0.010 to 1.00 ng/mL (R^2^ = 0.993) and the RSDs was from 2.91% to 11.3%. The linear response is a property of the utmost importance for implying feasibility of the established platform.

Sample matrix problem is a barrier in real sample analysis. The problem not only has effect on sensitivity but also on the analytical accuracy. It can be seen that the recoveries and reproducibilities are decreased in the real samples in Table 2. Since drink water often contains lots of metal ions, we deduced that the metal ions enable to alter the luminescent of CNPs and weaken interaction among OTC, CNPs, and O3. Then, TC was used as competitor and different kinds of buffer were studied, and TE buffer are more useful for improving the sensitivity and the reproducibility. Finally, high recoveries and lower RSDs were obtained, revealing that sample matrix was effectively reduced and the accuracy of assay was guaranteed.

In summary, we have synthesized and characterized a novel CNPs probe, and demonstrated its potential application for ssDNA based luminescent switch-on detection of OTC. The quenching of the CNPs luminescence by the utility of OTC is restored in the presence of ssDNA due to the specific formation of stable OTC-ssDNA complex. This system displays a strong luminescence “switch-on” response to OTC with a low limit of detection. Furthermore, the system exhibits a good linear range of OTC detection. The established method is simple, rapid and cost-effective. Although CNPs have been extensively utilized and lots of analytical methods for OTC is developed, this is the first to use a ssDNA based-CNPs as a probe for the detection of OTC by using OTC as luminescent quencher. We envisage that the luminescence CNPs could be further developed as antibiotic residues for the sensitive detection of antibiotic residues in aqueous solution.

## Methods

### Preparation of CNPs

C-Dots were prepared by the microwave method. Briefly, 15 mL of glycerine and 3 mL/g of ethylenediamine were mixed to become a homogeneous solution. Then, 1.0 g of citric acid was added into the solution and the mixture was heated in a microwave oven for 5 min. After that, 1.5 mL 85% phosphate solution was added to the above solution and heated 2 min. After cooling to room temperature, it was dispensed into 10 mL centrifuge tubes and centrifuged at 10,000 rpm, the centrifugal force was equal to 9305 g. The supernatant liquid was purified by dialysis and the cutoff of the dialysis membrane was equivalent to Mw≈500 Da.

### CNPs with ssDNA probe for OTC assay

Seven kinds of drink water were purchased from local super-markets. After the pH value of the water sample was adjusted to 7.4 with NaOH or HC1. Then, 100 μL sample was added into 2 μL CNPs solution with 100 μg/mL. After that, 88 μL TE buffer were injected. After shaking for 1 min, 10 μL 1.0 μM DNA was added and continue to shake 1 min. After 10 min, the treatment water sample was analyzed by F-7000 fluorescence spectrophotometer and the wavelength of excitation light was set at 358 nm.

## Supplementary information

Supplementary Information
