# Supplementary material for: Carbon nanoparticles with oligonucleotide probes for a label-free sensitive antibiotic residues detection based on competitive analysis
Source: Sci Rep. 2019 Mar 5;9:3489. doi: 10.1038/s41598-019-40209-1 (PMC6401375; doi:10.1038/s41598-019-40209-1)
Supplement: Supplementary file 1 — Supplementary Information [file 41598_2019_40209_MOESM1_ESM.pdf]

Supplementary Information

Carbon nanoparticles with oligonucleotide probes for a label-free sensitive antibiotic residues detection based on competitive analysis

Xuexia Lin [1*], Jianlong Su[1], Honggui Lin[2], Shu-Feng Zhou[1], Xiangying Sun[3], Bin Liu[3], Mingrong Zeng[1]

[1]Depaertment of Chemical Engineering & Pharmaceutical Engineering, College of Chemical Engineering, Huaqiao University, Xiamen, 361021 China

[2]School of Marine Engineering, Jimei University, Xiamen, 361021 China

[3]College of Materials Science and Engineering, Huaqiao University, Xiamen, 361021 China

Materials

glycerine, ethylenediamine, citric acid, Tris (hydroxymethyl) methyl aminomethaneand, Ethylenediaminetetraacetic acid, Potassium Phosphate Monobasicall, Dibasic Sodium Phosphate were purchased from Sinopharm Group Chemical Reagent (Shanghai, China). 4-(2-Hydroxyethyl)-1-Piperazineethanesulfonicaci was purchased from Sigma-Aldrich. Standard products of oxytetracycline (OTC), chlortetracyclin (CTC), tetracycline (TC), chloramphenicol (CAP), streptomycin (SM) and ampicillin (AP) were purchased from Solarbio@ (Beijing, China). De-ionized water was used in all experiments. OTC aptamers (O1, O2, O3, O4 and O5)) were synthesized by Sangon Biological Engineering Technology & Services Co, Ltd. (Shanghai, China).

0.2 M CPBS buffer（pH=7.4）was prepared by 0.2 M dibasic sodium phosphate and 0.1 M citric acid. 50 mM Tris-EDTA buffer (pH=7.4) was prepared by 100mM Tris (hydroxymethyl) methyl aminomethaneand and 1mM ethylenediaminetetraacetic acid. 1 M HEPES buffer (pH=7.4) was prepared by 1M 4-(2-Hydroxyethyl)-1 piperazineethanesulfonicaci and 0.5 M sodium hydroxide. 0.01 M PBS buffer (pH=7.4) was prepared by 0.01 M dibasic sodium phosphate and 1.7 mM potassium phosphate monobasic.

Apparatus

Fluorescence spectra were measured on an F-7000 fluorescence spectrophotometer (Hitachi, Japan). Ultravioletvisible (UV-vis) absorption spectra were recorded by ultraviolet specteophotometer 2600 (Shimadzu,Japan). The size and morphology of thenanoparticles were observed on a JEM-2100HR transmission electron microscope (TEM; JEOL Ltd.,

Japan). Fourier-transformed infrared (FTIR) spectra were obtained using an FTIR spectrophotometer (Spectrum two; PerkinElmer, USA) .

FIGURE AND TABLE LEGENDS

Figure S1 pH effect on the luminescence of CNPs.

Figure S2 Different concentrations of OTC and TC effect on the luminescence of CNPs.

Figure S3 Different kind of ssDNA influence on the different concentration of OTC analysis.

Table S1. Summary of other methods in recent years to detect oxytetracycline.

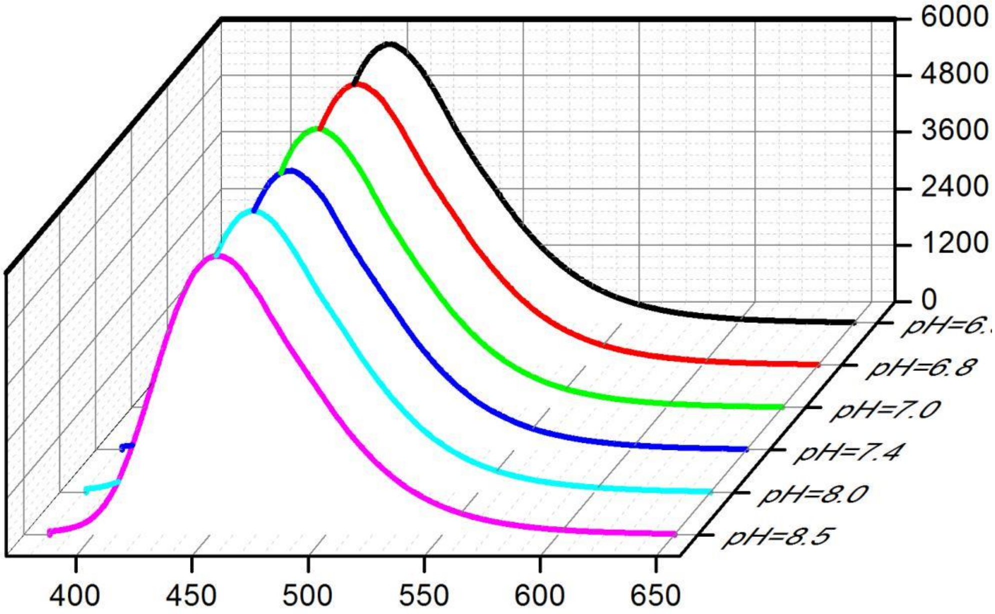
6000
4800
3600
2400
1200
0
pH=6.
pH=6.8
pH=7.0
pH=7.4
pH=8.0
pH=8.5
400
450
500
550
600
650

Fig.S1

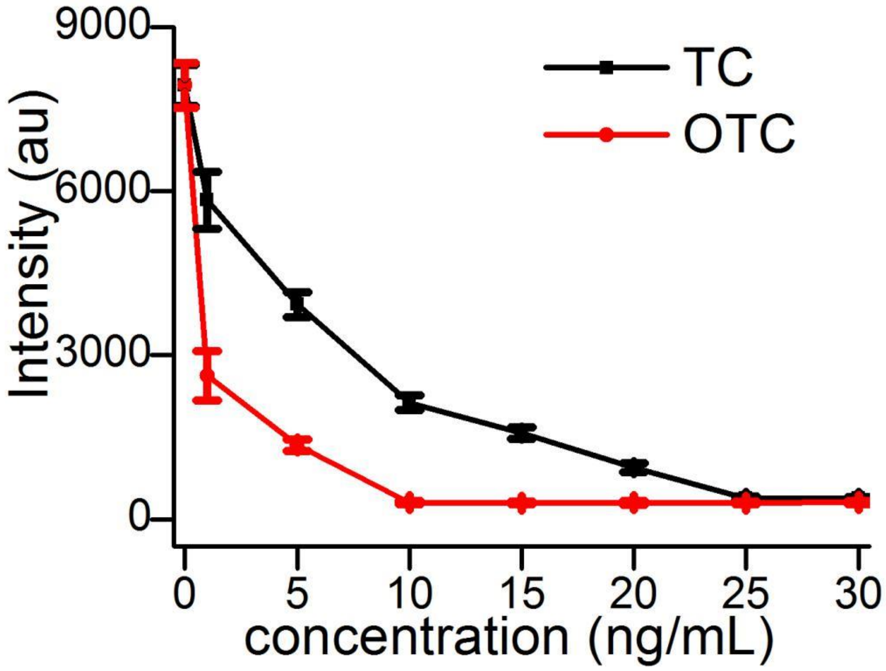
9000
6000
3000
0
Intensity (au)
TC
OTC
0
5
10
15
20
25
30
concentration (ng/mL)

Fig.S2

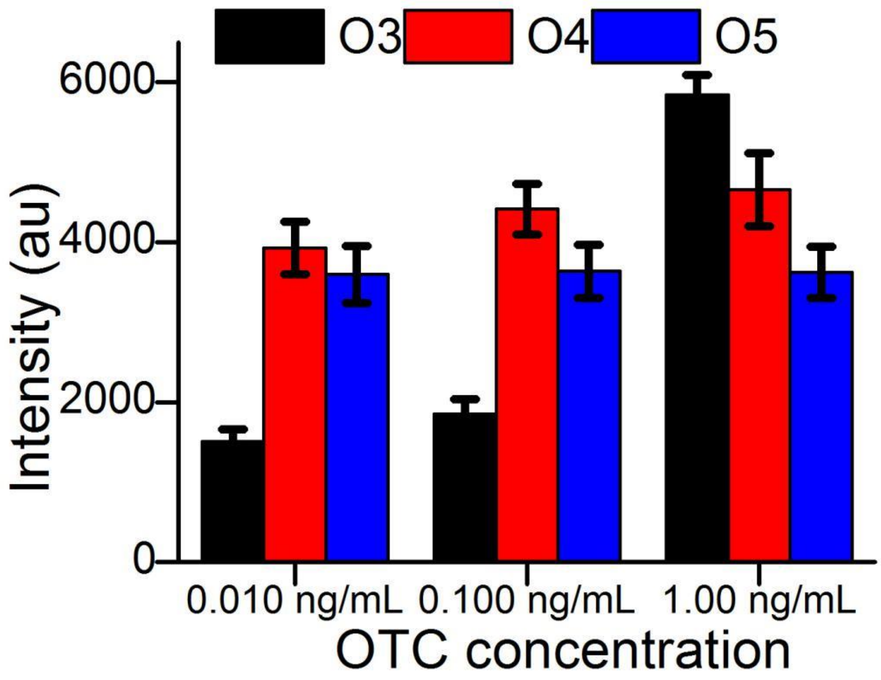
O3
O4
O5
6000
4000
2000
0
Intensity (au)
0.010 ng/mL
0.100 ng/mL
1.00 ng/mL
OTC concentration

Fig.S3

Table S1

Summary of other methods in recent years to detect oxytetracycline

Assay	Indicator or amplication	Linearity range	LOD	Ref.
Fluorescent Assay	BODIPY-based fluorescent probe	0 uM to 42 uM	68.1 nM	1
Fluorescent Assay	carbon dots and Fe_3O_4 MNPs	25 nM to 1.75uM	9.5 nM	2
Fluorescent Assay	silver nanoclusters	0.5 nM to 100 nM	0.1 nM	3
Fluorescent Assay	silicon-based nanoparticles	0.2 μM to 20 μM	0.18 uM	4
Photo-electrochemical	Grapheme, p-type semiconductor BiOI	4 nM to 150 nM	0.9 nM	5
Electrochemical	gold nanocomposite, horseradish peroxidase	0.1 nM-4 uM	0.01 nM	6
Colorimetric	Grapheme, gold nanoparticle	0.17 μM to 0.50 μM	91 nM	7
Electrochemical	Cantilever Array	0.1 nM-100 nM	0.2 nM	8

REFERENCES

1. Xu, Z., Yi, X., Wu, Q., Zhu, Y., Ou, M., Xu, X. First report on a BODIPY-based fluorescent probe for sensitive detection of oxytetracycline: application for the rapid determination of oxytetracycline in milk, honey and pork. RSC. ADV. 6, 89288-89297 (2016).
2. Wang, Y. *et al.* Highly sensitive fluorometric determination of oxytetracycline based on carbon dots and Fe3O4 MNPs. Sensor. Actual. B-Chem. 254, 1118-1124 (2018).
3. Hosseini, M., Mehrabi, F., Ganjali, M.R., Norouzi, p. A fluorescent aptasensor for sensitive analysis oxytetracycline based on silver nanoclusters. Luminescence 31, 1339-1343 (2016).
4. Xu, N., Yuan, Y.Q., Yin, J.H. Wang, X., Meng, L. One-pot hydrothermal synthesis of luminescent silicon-based nanoparticles for highly specific detection of oxytetracycline via ratiometric fluorescent strategy. RSC. ADV. 7,48429-48436 (2017).
5. Yan, K., Liu, Y., Yang, Y.H., Zhang, J.D. A Cathodic “Signal-off” Photoelectrochemical Aptasensor for Ultrasensitive and Selective Detection of Oxytetracycline. Anal. Chem. 87, 12215-12220 (2015).
6. Liu, S., Wang, Y., Xu, W., Leng, X.Q., Wang, H.Z., Gao, Y.N. A novel sandwich-type electrochemical aptasensor based on GR-3D Au and aptamer-AuNPs-HRP for sensitive detection of oxytetracycline. Biosens. Bioelectron. 88, 181-187 (2017).
7. Yuan, F., Zhao, H.M., Wang, X.D., Quan, X. Determination of Oxytetracycline by a Graphene-Gold Nanoparticle-Based Colorimetric Aptamer Sensor. Anal. Lett. 50, 544-553 (2017).
8. Hou, H., Bai, X.J., Xing, C.Y., Gu, N.Y., Zhang, B.L., Tang, J.L. Aptamer-Based Cantilever Array Sensors for Oxytetracycline Detection. Anal. Chem. 85, 2010-2014 (2013).
